
JOURNAL INFORMATION
==============================
NLM Title Abbreviation: ScientificWorldJournal
Journal ID: ScientificWorldJournal
NLM Journal ID: 101131163
Journal ID: TSWJ

The Scientific World Journal

ISSN: 2356-6140
EISSN: 1537-744X
Publisher: Wiley

ARTICLE INFORMATION
==============================
PMCID: PMC6084127
PMID: 30147615
DOI: 10.1100/tsw.2001.235
Article ID: 917610
Article version: 1
Subjects: Short Report

Genetic Control of Programmed Cell Death in C. Elegans

Horvitz H. Robert *
Howard Hughes Medical Institute, Department of Biology, Massachusetts Institute of Technology, Cambridge, MA 02139, USA
*H. Robert Horvitz: horvitz@mit.edu
Publication date: 2001
Electronic publication date: 2001 Aug 17
Volume: 1
First page: 137
Last page: 137
Copyright: Copyright © 2001 H. Robert Horvitz.
Copyright year: 2001
License: This is an open access article distributed under the Creative Commons Attribution License, which permits unrestricted use, distribution, and reproduction in any medium, provided the original work is properly cited.
License URL: https://creativecommons.org/licenses/by/3.0/

==============================
Miami *Nature Biotechnology* Short Reports
*TheScientificWorld* (2001) 1 (S3), 137SR
ISSN 1532-2246; DOI 10.1100/tsw.2001.235

### GENETIC CONTROL OF PROGRAMMED CELL DEATH IN *C. ELEGANS*

H. Robert Horvitz

Howard Hughes Medical Institute, Department of Biology, Massachusetts Institute of Technology, Cambridge, MA 02139, USA
horvitz@mit.edu

Apoptosis or programmed cell death is a major feature of the normal development of the nervous system. The clinical features of a variety of neurological disorders – including neurodegenerative diseases, stroke and traumatic brain injury – are a consequence of excessive neuronal cell death. One current hypothesis is that much of this excessive cell death involves the same mechanisms as those used during normal nervous system development. I will discuss findings concerning the mechanisms of apoptosis based primarily upon our studies of the nematode *Caenorhabditis elegans*. During the development of *C. elegans,* 131 of the 1,090 cells generated undergo programmed cell death. More than 80% of these cell deaths are in the nervous system. We have characterized developmentally, genetically and molecularly the roles of 15 genes that function in *C. elegans* programmed cell deaths. Six of these genes control the death process, six act in the phagocytosis of dying cells by their neighbors, and one functions in the digestion of the DNA of cell corpses; two additional genes specify which cells will or will not express this cell-death program. Many of these genes show structural and functional similarities to genes that act in mammalian apoptosis, indicating that the mechanisms of apoptosis are conserved among organisms as distinct as nematodes and humans. Our studies have led to a model for the developmental activation of programmed cell death in *C. elegans*: increased transcription of the *egl-1* gene elevates the level of the EGL-1 protein, which is similar in sequence to the BH3-domain-containing cell-death killer proteins of mammals. The EGL-1 protein binds to a complex consisting of at least the CED-9 protein (similar to human Bcl-2) and the CED-4 protein (similar to human Apaf-1), releasing CED-4 and allowing it to activate the CED-3 caspase by promoting the proteolytic cleavage of a CED-3 proprotein.
